# Indispensable Nafion Ionomer for High-Efficiency and Stable Oxygen Evolution Reaction in Alkaline Media

**DOI:** 10.1021/acsami.3c08377

**Published:** 2023-11-21

**Authors:** Nitul Kakati, Lawrence Anderson, Guangfu Li, Desiree Mae Sua-an, Ayon Karmakar, Joey D. Ocon, Po-Ya Abel Chuang

**Affiliations:** † Department of Mechanical Engineering, University of California, Merced, Merced, California 95343, United States; ‡ Foshan Xianhu Laboratory of the Advanced Energy Science and Technology, Guangdong Laboratory, Xianhu Hydrogen Valley, Foshan 528200, China; § Laboratory of Electrochemical Engineering, Department of Chemical Engineering, University of the Philippines Diliman, Quezon City 1101, Philippines

**Keywords:** oxygen evolution reaction, TF-RDE/RRDE, catalyst layer structure, Nafion ionomer, drop-casting

## Abstract

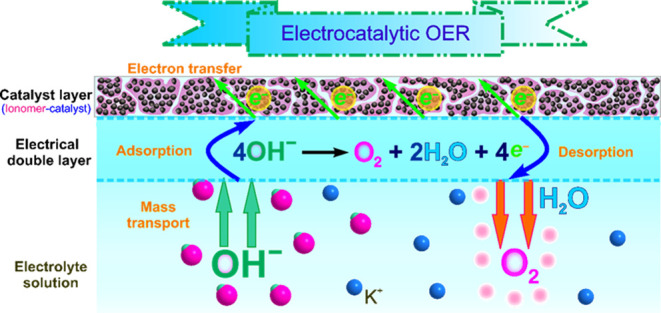

Addressing the challenge of sluggish kinetics and limited stability in alkaline oxygen evolution reactions, recent exploration of novel electrochemical catalysts offers improved prospects. To expedite the assessment of these catalysts, a half-cell rotating disk electrode is often favored for its simplicity. However, the actual catalyst performance strongly depends on the fabricated catalyst layers, which encounter mass transport overpotentials. We systematically investigate the role and sequence of electrode drop-casting methods onto a glassy carbon electrode regarding the efficiency of the oxygen evolution reaction. The catalyst layer without Nafion experiences nearly 50% activity loss post stability test, while those with Nafion exhibit less than 5% activity loss. Additionally, the sequence of application of the catalyst and Nafion also shows a significant effect on catalyst stability. The catalyst activity increases by roughly 20% after the stability test when the catalyst layer is coated first with an ionomer layer, followed by drop-casting the catalysts. Based on the half-cell results, the Nafion ionomer not only acts as a binder in the catalyst layer but also enhances the interfacial interaction between the catalyst and electrolyte, promoting performance and stability. This study provides new insights into the efficient and accurate evaluation of electrocatalyst performance and stability as well as the role of Nafion ionomer in the catalyst layer.

## 1 Introduction

The rising energy demand and depleting fossil fuel supplies have shifted global attention to renewable and sustainable energy technologies, including hydrogen energy as a clean alternative fuel.^1^ However, the technologies for producing hydrogen from new and renewable energy sources are not yet matured, and the cost of production remains very high and uncompetitive compared with that generated from fossil fuels. Currently, around 95% of generated hydrogen originates from carbonaceous raw materials, mostly of fossil origin, with just 4% generated by water electrolysis.^2^ In comparison to acidic electrolysis, liquid alkaline electrolysis is a more well-developed technology. Alkaline electrolysis is less costly as it employs non-noble-metal electrocatalysts such as Ni, Co, Fe, Mn, and Mo.^3−8^ Additionally, compared to that in the alkaline medium, significant corrosion can be observed in an acidic medium.

In water electrolysis reactions, the oxygen evolution reaction (OER) is much more sluggish compared with the hydrogen evolution reaction (HER) due to slow kinetics. Therefore, considerable research effort has been made to enhance the activity of the OER by using different catalyst materials. The need for noble-metal catalysts, such as IrO_*x*_ and RuO_2_, as well as their poor kinetic performance, are primary challenges in proton exchange membrane water electrolysis.^9,10^ When water electrolysis is performed in alkaline media, the use of noble-metal catalysts can be reduced or eliminated entirely. With the rising interest in alkaline anion exchange membrane water electrolysis, there is a greater need for more sophisticated and less costly OER catalysts.^11−14^ In the past few decades, research and development on the activity and long-term stability researches have resulted in substantial improvements in OER catalysts.^15−18^ However, correctly assessing actual catalyst performance in alkaline media remains a challenge.^19−22^ The results from a three-electrode system based on a thin film rotating disk electrode (TF-RDE) and thin film rotating ring disk electrode (TF-RRDE) are widely used to estimate catalyst performance in a full electrochemical cell.^23−29^ However, ambiguities arise in the majority of the TF-RDE/RRDE results due to the numerous process steps involved in ink production and deposition on the electrode surface, operating conditions, and multiple measurement methodologies. In their study, Riasse et al. compared various methods of catalyst evaluation, including RDE, gas diffusion electrode, and differential cell.^30^ Each method exhibited its own set of advantages and drawbacks. In specific, the RDE study demonstrated high mass transport limitations but offered a clean experimental environment and ease of operation and notably required only a small quantity of catalyst for the investigation.

Nafion ionomer is commonly employed in TF-RDE/RRDE studies as a dispersion for catalyst particles in catalyst ink as well as a binder to prevent the catalyst layer (CL) from peeling off the electrode surface.^31−33^ The CL coated on the electrode surface is known to have a significant impact on the electrochemical performance.^34^ The key parameters that influence electrochemical characteristics are typically linked to the morphology of the catalyst ink, the coating technique, and the drying processes.^35,36^ In diverse electrochemical processes, such as proton exchange membrane fuel cells (PEMFCs) and anion exchange membrane fuel cells (AEMFCs), several authors have described the relevance of the ionomer in the catalyst ink with an optimal ionomer-to-catalyst ratio.^37−40^ Due to the absence of contrast between Nafion and the catalyst particles, imaging the CL under a state-of-the-art electron microscope to obtain information on Nafion morphology and transport behavior is very challenging.^41^ As a result, electrochemical techniques have attracted more attention as an effective method to investigate and understand the CL’s interfacial mechanism and electrochemical behavior, revealing the function of the ionomer in the CL.

In a recent research, we employed in situ and ex situ methods to analyze the critical function of Nafion and provide new insights into how the presence of the Nafion ionomer in the CL affects the electrical double layer (EDL) structure.^24^ The importance of the ionomer in the alkaline OER is a crucial but relatively understudied aspect. The ionomer plays a significant role in determining the properties of the ink and the physical and electrochemical behaviors of the catalyst layers (CLs), particularly at the reaction EDL interface. In our recent study, the CL containing Nafion exhibited the highest OER activity in the alkaline medium compared to those prepared using the anion exchange ionomer (AEI) As-4 (Tokuyama Corp.) and the nonionic PTFE binder (Fuel Cell Store).^24^ Lu et al. conducted research on an imidazolium-functionalized poly(ether sulfone) as an AEI for use in fuel cells. They compared its electrochemical characteristics with those of AS-4 and Nafion ionomers.^42^ In their study, the researchers also found that the Pt/C catalyst using the Nafion ionomer showed the highest mass activity. Even though the goal for alkaline OER is to eliminate the use of precious metal and perfluoroalkyl substances (PFAs), Nafion is still the most commonly used in preparing catalyst ink for evaluating electrocatalyst activities. Therefore, in the present study, we have expanded our investigation to study the influence of Nafion and CL coating processes on the activity and stability of the OER in an alkaline medium. The objective of this investigation is to develop a fundamental understanding of how the Nafion ionomer in catalyst inks affects the CL and impacts the OER mechanism of the IrO_*x*_ catalyst.

## 2 Experimental Section

### 2.1 Chemical Reagents

Potassium hydroxide (KOH, ≥90 wt %), anhydrous ethanol (≥95 wt %), and 1 wt % Nafion solution were purchased from Sigma-Aldrich. Iridium oxide (IrO_*x*_, 99.99%) was obtained from Alfa Aesar. Solutions were prepared by using ultrapure deionized water (18 MΩ cm resistivity) supplied by an in-house water purification system from Thermo Scientific Barnstead Nanopure.

### 2.2 Structural Characterization

The Zeiss Gemini field emission scanning electron microscope (FESEM) 500 operating at 5 kV was used to capture the SEM images. The IrOx-based catalyst ink was drop-cast onto a glassy carbon electrode (GCE) for all SEM samples.

### 2.3 Electrochemical Tests

A bipotentiostat (CHI 750E) was used to conduct the electrochemical tests at room temperature and ambient pressure. A Hg/HgO electrode (filled with 1 M KOH), a platinum wire (7 mm OD × 65 mm), and an RDE/RRDE were used as the reference electrode (RE), counter electrode (CE), and working electrode (WE), respectively, in a standard three-electrode system. The RDE (E3, Pine Instrument Co.) had a GCE with an outer diameter (OD) of 5 mm, and the RRDE (E7R9, Pine Instrument Co.) had a submillimeter gap between its GCE disk (5.61 mm OD) and platinum ring (7.92 mm OD, 6.25 mm ID). The calibrated potential of the applied Hg/HgO reference electrode vs the reversible hydrogen electrode (RHE) in 1 M KOH at room temperature was found to be 0.898 V. A 300 mL PTFE cell filled with N_2_-saturated 6 M KOH was used to immerse the three-electrode system. To examine the influence of catalyst configuration on the electrochemical behavior of the OER, the catalyst ink was deposited over the GCE in four distinct ways. In the first mode, the catalyst ink was prepared by dispersing 5 mg of IrO_*x*_ in 4 mL of ethanol through sonication and drop-cast without the use of Nafion electrolyte (No-I); in the second mode, it was prepared by combining catalyst powder (IrO_*x*_), ethanol, and 1% Nafion ionomer (Mix-I); and in the third mode, a drop of 1% Nafion ionomer was cast first, and then after drying the ionomer under an IR lamp, catalyst ink prepared without mixing ionomer was drop-cast over the Nafion ionomer layer. In the fourth mode, the processes in the third mode were reversed; i.e., the CL was deposited first, and the ionomer layer was deposited over the CL.

The standard catalyst loading was maintained at 0.08 mg cm^–2^ throughout the experiment. We also investigated the influence of catalyst loading on the electrochemical performance by using a higher loading of 0.24 mg cm^–2^. In addition, to investigate the configurational influence of CLs on electrochemical characteristics, we synthesized CLs with three layers, such as ionomer-catalyst-ionomer (I–C–I) and catalyst-ionomer-catalyst (C–I–C), and compared their catalytic efficiency toward OER with their two-layer equivalent. In all CLs, the catalyst-to-ionomer ratio was kept constant. Cyclic Voltammetry tests were performed under static conditions (rotating speed = 0), and a standard electrode rotating speed of 1600 rpm was used while performing the linear scanning voltammetry (LSV) and electrochemical impedance spectroscopy (EIS) to facilitate mass transport and O_2_ removal from the reaction interface. For accelerated stability evaluation, pulsed chronoamperometry was used with a pulse width of 30 s. The potentials were pulsed at 1.45 and 1.55 V vs RHE and completed 60 steps in 1800 s and the LSV and EIS were compared with those before the stability tests.

The effect of the ink coating technique on the EDL charging process and the efficiency of transporting the O_2_ during the OER was demonstrated using TF-RRDE experiments. Standard voltammetry curves were obtained at a rotating speed of 1600 rpm while the disk potential was increasing with time and the ring potential was held at 0.4 V for oxygen reduction reaction. The geometric collection efficiency of the RRDE was measured to be 37.3% by detecting the redox couple [Fe(CN)_6_]^3–^/[Fe(CN)_6_]^4–^. To evaluate the ring collection efficiency for a gas-evolving reaction, hydrogen evolution has been chosen as a model reaction at the disk electrode with a commercial Pt/C catalyst in accordance with Chung et al.^43^ The catalyst ink was prepared by dispersing 3 mg of commercial Pt/C (46.8%) powder in 2 mL of ethanol and 27 μL of Nafion solution (5 wt %) for 30 min under sonication. To prepare the working electrode, 40 μL of the resultant ink was drop-cast only on the glassy carbon (GC) disk of an RRDE electrode with the Pt-ring. After being dried under the IR lamp, LSV curves were recorded by the RRDE in N_2_-saturated 0.1 M KOH by sweeping the potential from 0.1 to −0.23 V vs RHE at the GC-disk for HER and the ring potential was held at 0.2 V vs RHE. The collection efficiency was calculated from the ratio of the absolute value of the current responses at the ring to disk and found to range between 40% (low current) and 18% (high current) as shown in Figure S12. The collection efficiency of the realistic hydrogen evolution reaction stabilizes at around 18.1%, which is much less than the geometric collection efficiency obtained from the redox couple [Fe(CN)_6_]^3–^/[Fe(CN)_6_]^4–^ mainly due to bubble formation.

## 3 Results and Discussion

**Figure 1:**
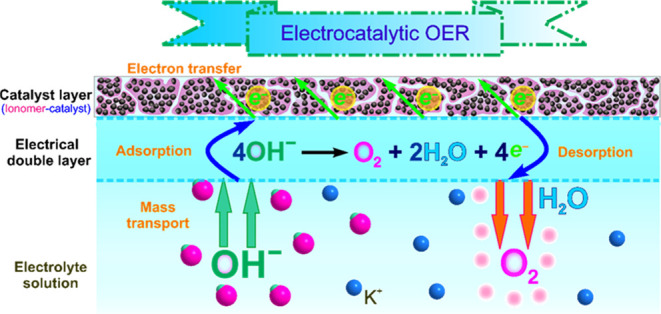
Schematic illustration of the interfacial OER with emphases on the mass transport behaviors in alkaline media.

Oxygen evolution reaction involves complicated multisteps of electrochemical reactions occurring near the catalyst surface. Figure 1 presents a schematic depiction of the reaction process at the EDL area, which constitutes the interface between the catalyst surface and the electrolyte. At the applied OER potential, the Coulombic attraction between OH^–^ ions and CL causes the adsorption of OH^–^ ions on the active CL. The OER electrocatalysts layer facilitates the faradaic conversion of OH^–^ ions into electrons, water, and oxygen. The rate of the OER depends on the number of available active electrocatalyst sites, as well as the OH^–^ ions, electrons, and O_2_-transport resistances in the EDL.^44,45^

**Figure 2:**
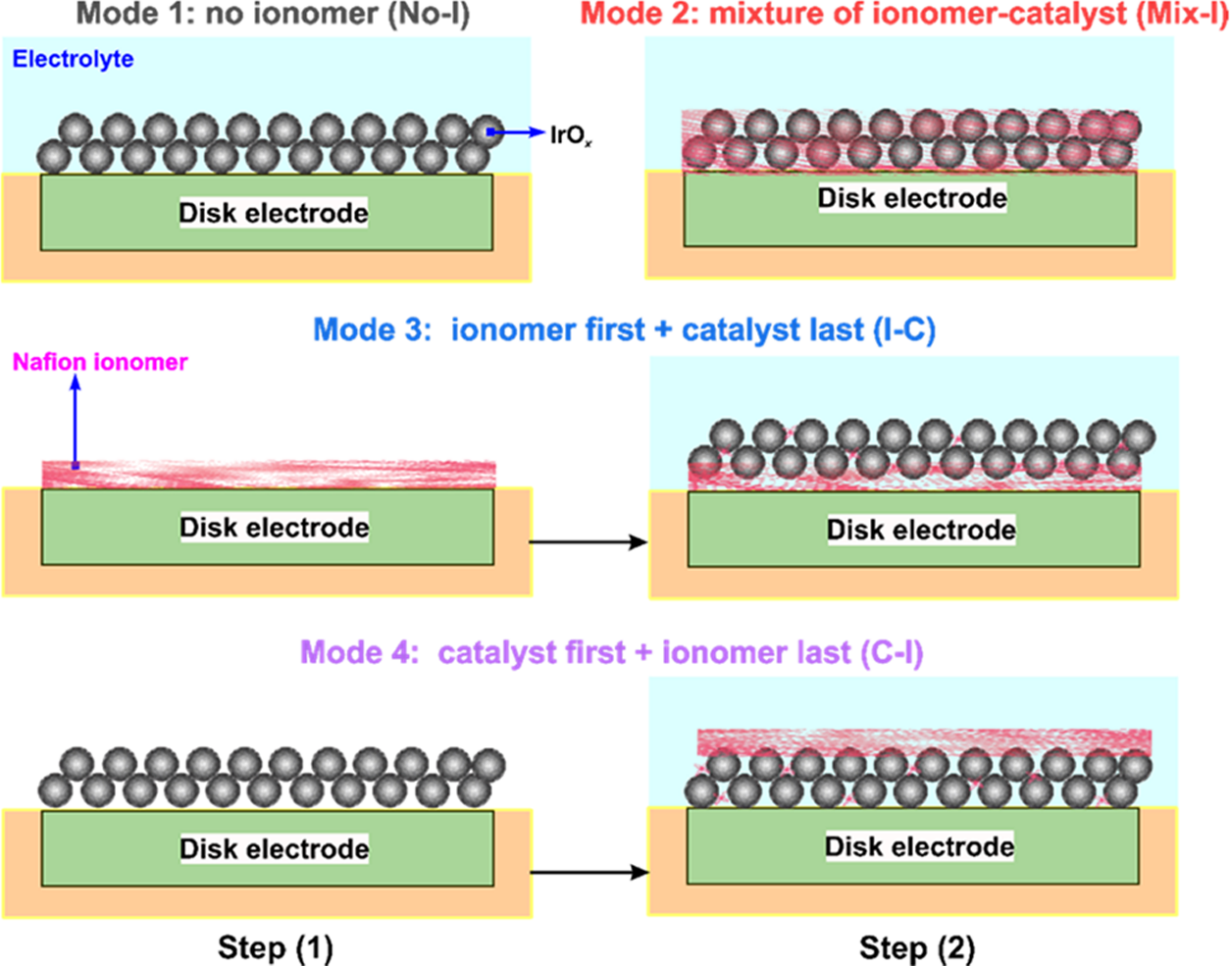
Illustration of the four Nafion ionomer deposition modes in the CLs for TF-RDE/RRDE evaluation.

The different modes of catalyst and Nafion deposition on the surface of a GCE are illustrated schematically in Figure 2. Under mode 1, the CL does not include the ionomer, while in mode 2, the catalyst ink is formulated with the ionomer. Mode 3 involves the deposition of a Nafion ionomer layer on the GCE, followed by the deposition and drying of the catalyst ink over the ionomer layer. In mode 4, the process is reversed, with the catalyst ink being deposited and dried on the GCE, followed by the application of a second layer of the Nafion ionomer. The presence of Nafion ionomer with CL can act as a binder, preventing catalyst loss and increasing the reaction interface. Additionally, our previous studies have shown that the ζ-potential of catalyst inks increases with the amount of Nafion ionomer in the ink, resulting in enhanced electrostatic repulsion between the catalyst agglomerates and improved ink stability.^24,27^

**Figure 3:**
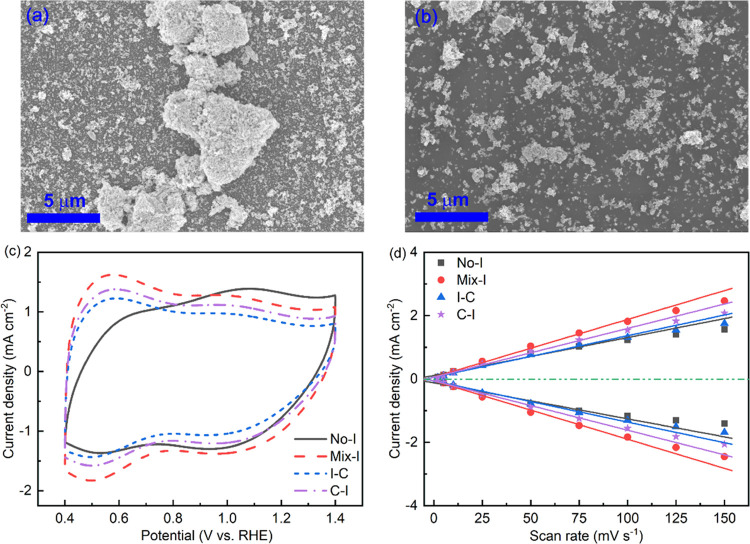
SEM images of the IrO_*x*_ CLs using inks (a) without ionomer and (b) with mixed ionomer. Electrochemical data of the (c) pseudocapacitive CV curves at 50 mV s^–1^ and (d) anodic (above zero) and cathodic (below zero) EDL capacitive current density at 0.9 V as a function of scan rate (symbols represent raw data and the lines are linearly fitted results in the scan rate range of 2–100 mV s^–1^).

SEM images of CLs on GCE without and with Nafion ionomer are presented in Figure 3a,3b, respectively. These images demonstrate the significant impact of the Nafion ionomer on the dispersion of the catalyst ink. Without the ionomer, the dispersion of the catalyst ink is negatively affected, leading to the formation of large particle aggregates, decreased active surface area, and reduced catalyst utilization. The CV curves reveal a distinctive “fingerprint” region in the potential range of 0.4–1.4 V for IrO_*x*_,^46^ as observed in Figure 3c. The current shown in Figure 3c encompasses both the EDL charging process and the pseudocapacitive current resulting from redox processes. To isolate the influence of pseudocapacitance, CV curves in Figure S1 were derived from Figure 3c within the potential range of 0.8–1.0 V. The rectangular-like shape of the CV curves in Figure S1 confirms the pure non-Faradaic capacitive behavior.^47^Figure 3d displays the experiment measurements of single-point anodic and cathodic currents at 0.9 V for various scan rates ranging from 2 to 150 mV s^–1^ and linearly fitted line over a scan rate range of 2–100 mV s^–1^. The slopes of the linear fitting of the single-point currents at different scan rates provide information about the EDL capacitance and, subsequently, an estimate of the trend in the electrocatalytic surface area (ECSA) of the CLs. From Figure 3d, the ECSA of the CLs decreases in the following order: Mix-I > C–I > I–C > No-I. In a previous study, we demonstrated that the ECSA calculated using the EIS approach is more accurate than the CV method.^25^ Therefore, in this study, we analyzed the ECSA using the EIS approach and compared the trend to the CV method.

**Figure 4:**
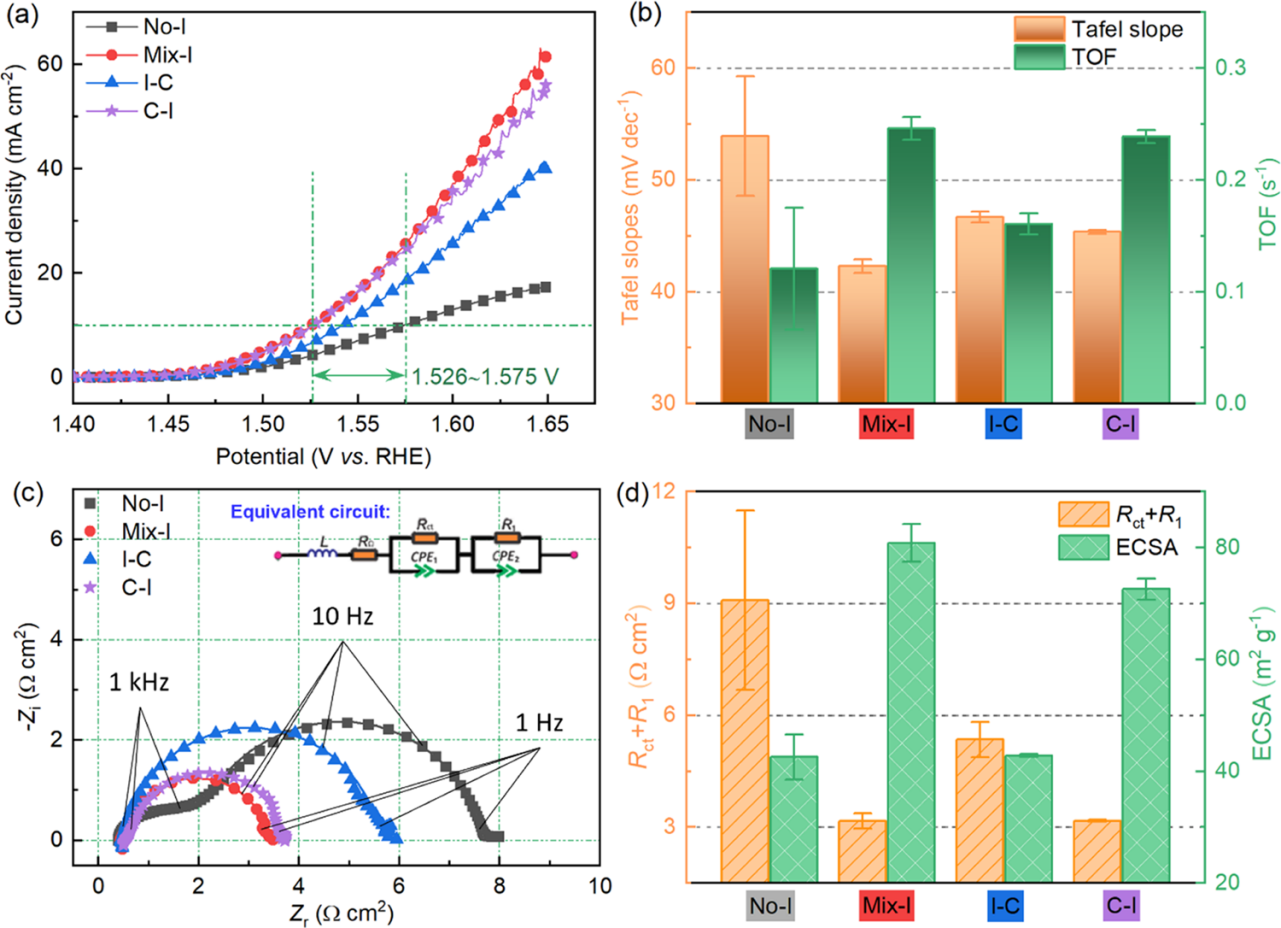
Electrocatalytic activity evaluation as a function of ionomer coating modes. (a) LSV curves at 2 mV s^–1^, (b) Tafel slopes fitted between 0.3 and 3 mA cm^–2^ and TOF at *iR*-corrected voltage of 1.58 V, (c) EIS spectra at 1.55 V, the inset is the equivalent circuit for EIS simulation (symbols: raw data; lines: simulation results), and (d) the calculated total polarization resistance (*R*_ct_ + *R*_1_) and ECSA. Error bars represent standard deviations from two independent measurements.

The electrocatalytic activity of the various CLs was evaluated by obtaining LSV curves at a scan rate of 2 mV s^–1^. Figure 4a shows the LSV curves obtained from TF-RDE experiments at a rotating speed of 1600 rpm. CLs coated with Nafion ionomer had a significantly lower overpotential in the kinetic region (10 mA cm^–2^) compared with CLs without Nafion (No-I). The overpotential for No-I increased by approximately 47 mV compared to Mix-I and C–I. Mix-I and C–I had overpotentials that were similar to, but lower than, No-I and I–C. It is important to note that the overpotential is influenced by both the kinetic and mass transport effects in the high-current-density region. No-I and I–C showed a larger increase in overpotential in the high-current-density region compared to Mix-I and C–I. Despite having identical overpotentials, C–I performed slightly worse in the high-current-density zone, possibly due to mass transport limitations imposed by the Nafion ionomer layer on top of the CL.^24^ In Figure S2, the SEM image of the C–I mode shows the ionomer layer covering the catalyst particles.

To further evaluate the OER performance of the CLs, *IR*-free turnover frequency (TOF) and Tafel plots were analyzed based on the Tafel slopes (Figures 4b and S3). The OER is widely recognized as a four-electron transfer process, encompassing a series of stages and numerous intermediate species like MO, MOOH, or physically adsorbed peroxide species.^48^ Over the course of many years, several potential pathways have been suggested for the mechanism. Among these, the Krasil’shchikov’s path mechanism^25^ holds prominence, which is outlined below with their corresponding Tafel slopes.
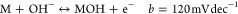
1
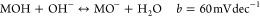
2
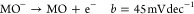
3
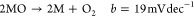
4

The Tafel slopes in Figure 4b are obtained from the transport-free reaction kinetics data in the region of 0.3–3 mA cm^–2^ scanned at 2 mV s^–1^ to ensure minimal capacitive current (see also Figure S3b). The Tafel slope, which is close to 45 mV dec^–1^ for CLs containing Nafion, indicates the formation of surface-adsorbed *O intermediate.^25,26^ However, No–I exhibited a substantially larger Tafel slope in the kinetic region. This phenomenon can be ascribed to the increase in bond strength of OH^–^ adsorption on catalysts containing Nafion, leading to a faster progression of the initial electron reaction depicted in eq 1, thereby enhancing electrocatalytic kinetics.^48^ On the other hand, the change in Tafel slopes can also reflect the concentration of active sites and their contribution.^25^ Therefore, it is likely that in this kinetic region, the thin Nafion layer covering the catalyst surface promotes the OER. In the high-current-density region (10–30 mA cm^–2^), the Tafel slopes of the CLs were significantly higher than those in the low-current-density region. The elevation in the Tafel slope can be ascribed to four underlying mechanisms: (i) enhanced resistance to mass transport of OH^–^ ions, electrons, and/or O_2_ molecules, (ii) alteration in the rate-determining step (RDS), (iii) adsorption of intermediates within the reaction, and (iv) modification of active sites and their involvement due to EDL reconstruction. The formation of O_2_ bubbles obstructing the accessible reaction surface results in additional resistance to mass transport.^49,50^ This hinders the adsorption of OH^–^ ions and the transport of electrons within the EDLs. To effectively mitigate the presence of O_2_ bubbles and enhance mass transport, a frequently employed strategy is to raise the rotational speed of the WE. In our previous study,^25^ we observed that elevating the rotational speed from 500 to 1600 rpm led to a marginal improvement in the OER performance. Mix-I had a TOF that was almost double that of No–I, suggesting that the presence of Nafion enhances both the interconnection among catalyst particles and the intrinsic activity toward OER.

The EDL during the OER is influenced by complex processes such as charge accumulation, transfer, and dissipation.^51^ The interfacial behaviors in the EDL were evaluated by using Electrochemical Impedance Spectroscopy (EIS) as an in situ characterization tool. The EIS spectra were collected at an applied DC potential of 1.55 V. The modeled equivalent circuit and simulated Nyquist plots from EIS are presented in Figure 4c. Different CLs exhibited significant variations in impedance, indicating that the catalyst drop-casting modes play an important role in electron transport, reaction polarization resistance, and EDL capacitive behaviors. The values of various impedance fitting parameters based on the EIS equivalent circuit are provided in Table S1. In the inset of Figure 4c, the equivalent circuit *LR*_Ω_(*R*_ct_*C*_1_)(*R*_1_*C*_2_) is used to fit and quantify the fitting parameters in the EIS spectra. In the equivalent circuit *L*, *R*_Ω_, *R*_ct_, *C*_1_, *R*_1_, and *C*_2_ represent inductance, ohmic resistance, charge transfer resistance, EDL charging capacitance, intermediate diffusion/adsorption resistance, and capacitance, respectively.^25^ In the equivalent circuit, two time-constant processes: charge transfer process and diffusion process in porous media are represented by *R*_ct_*C*_1_ and *R*_1_*C*_2_, respectively.^52,53^ To simulate nonideal capacitances arising from heterogeneity, surface porosity, and EDL reconstruction, a constant phase element (CPE) is used, such as *C*_1_ and *C*_2_.^54,55^^54,55^ The simulated results are consistent with the experimental data demonstrating the accurate representation of the OER process occurring in the EDL.

The ohmic resistance (*R*_Ω_), total polarization resistance (*R*_ct_ + *R*_1_), and electrochemical surface area (ECSA) were calculated by using the EIS simulated data (Table S1). The trends in total polarization resistance and ECSA as a function of CL casting modes are listed in Figure 4d. A detailed derivation of the ECSA from EIS data is reported in our previous study.^25^ Higher ECSA is associated with lower total polarization resistance, indicating that the interaction of the Nafion ionomer with catalyst particles plays a crucial role in the OER performance. Although No-I and I–C have identical ECSAs, I–C exhibits a substantially lower total polarization resistance. The presence of the Nafion ionomer in I–C is believed to contribute to a reduction in charge transfer resistance, intermediate diffusion, and adsorption during OER.

**Figure 5:**
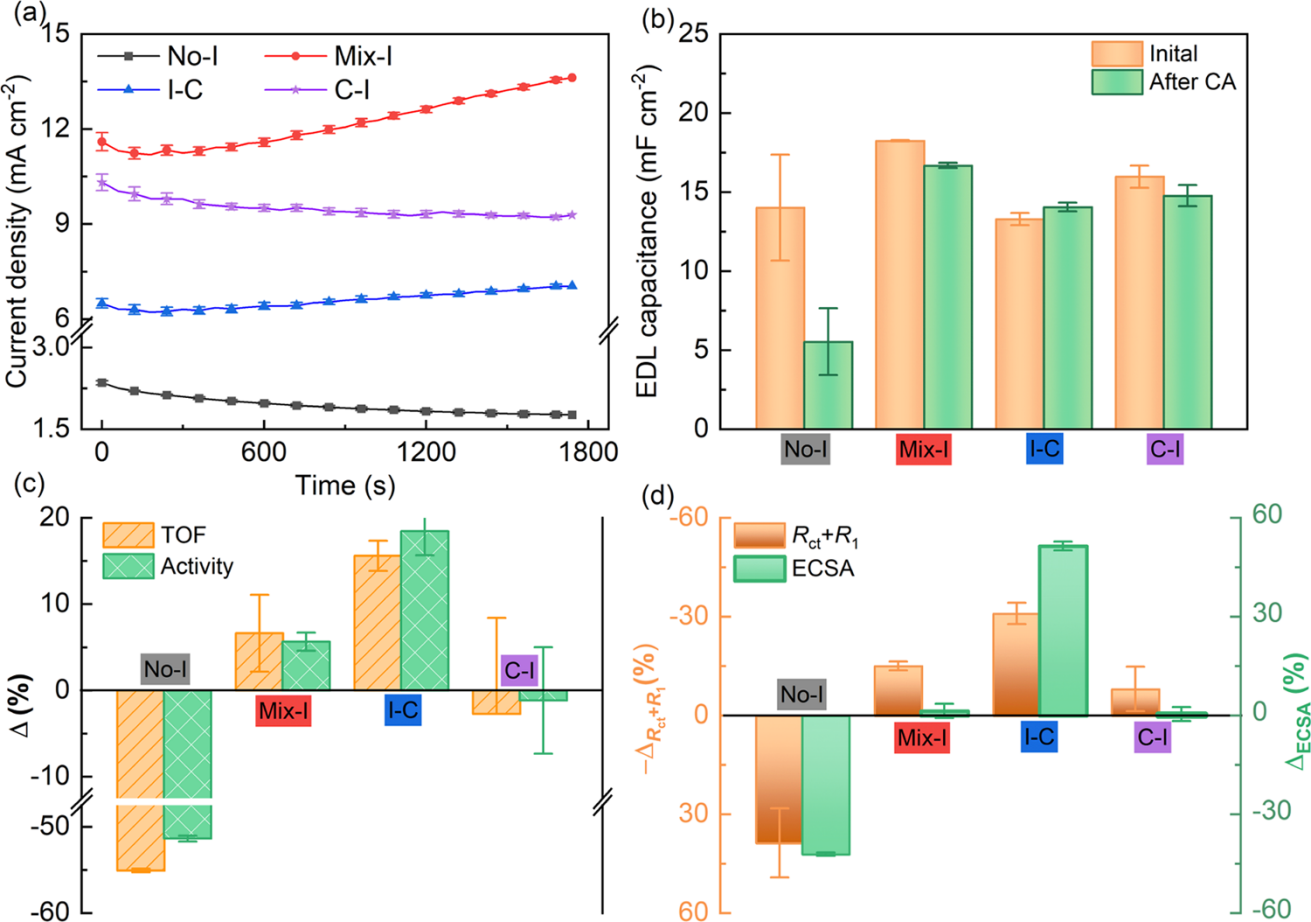
OER stability evaluation of IrOx by chronoamperometry (CA) measurements for different ionomer coating modes. (a) Current density at 1.55 V as a function of time, (b) EDL charging capacitance obtained by the single-point CV current method before and after CA, (c) change in TOF at *iR*-corrected voltage of 1.58 V and OER activity via LSV integrated area before and after CA, and (d) change of the total polarization resistance and ECSA before and after CA. To emphasize the performance loss, the “–” in “Δ*R*_ct_ + *R*_1_” is used to represent the decrease of reaction resistance.

The stability of the catalyst was assessed in 6 M KOH for 30 min using CA and a double-potential holding steps technique. Figure S4 depicts the detailed potential profile, which includes a high potential (1.55 V) step followed by a low potential step (1.45 V). El Sayed et al.^49^ conducted an investigation into catalyst degradation caused by micro bubble formation on the catalyst layer. They observed that the catalyst layer remained stable under low current density (5.5 mA cm^–2^) for several hours, while at high-current-density (27.5 mA cm^–2^) operation, the stability reduced to about 30 min. As found in their study, controlling the electrode at the open circuit potential (OCP) and subsequently purging the RDE setup with argon (Ar) allows the dissolution and diffusion processes to effectively eliminate the O_2_ bubbles. In our study, we assume that during the 30 s hold period at a low potential, the O_2_ generated during the OER at the high potential can be efficiently eliminated. The average current densities at the high potential steps for each CL are displayed in Figure 5a. The performance of the CLs was assessed by comparing changes in key parameters such as the TOF, the OER activity, *R*_ct_ + *R*_1_, and the ECSA shown in Figure 5. The comparison results were calculated as follows:
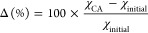
where χ_CA_ is the measured parameter after CA test and χ_initial_ is the initial measured parameter. Figure S5 presents various EDL capacitive behaviors of the CLs obtained from their CV curves. After the CA test, the EDL capacitance of the CLs was measured by using CV tests at various scan speeds (Figure S6) and compared to their initial EDL capacitance (Figure 5b). In Figure S5c, the ratio *C*_outer_/*C*_total_ represents the apparent porosity in the CL.^56^ The decrease in *C*_outer_/*C*_total_ observed in the stability test for the CLs with Nafion can be attributed to particle agglomeration and EDL reconfiguration during the OER process. After the stability test, the *C*_outer_/*C*_total_ increased for No-I, indicating the loss of catalyst particles without a binder in the CL. Figure S7 shows the SEM images of the No-I CL before and after the stability test. In Figure S7b,c, catalyst loss from the No-I CL can be clearly observed in the marked area. Figure S5d shows the interfacial reversibility, represented by the ratio of *C*_EDL_^+^/*C*_EDL_^–^, during the CV process. For CLs containing Nafion, the ratio of *C*_EDL_^+^/*C*_EDL_^–^ remains around 1, showing good reversibility before and after the stability test. The *C*_EDL_^+^/*C*_EDL_^–^ ratio is found to be higher than 1 for No-I before and after the stability test, confirming a lower interfacial reversibility for No-I. It is also plausible to assume that the anodic-to-cathodic capacitance ratio is connected to catalyst dissolution.^57^ A higher ratio corresponds to higher anodic dissolution during the OER. The CLs without Nafion showed a greater ratio than the CLs with Nafion, indicating that Nafion is capable of successfully preventing catalyst dissolution. Figure S8 shows LSV, TOF, and Tafel plots, as well as variations in Tafel plots following the CA test. Figure 5b shows that No-I had a significant drop in EDL capacitance, while the other CLs containing Nafion ionomer had only a slight change in EDL capacitance following the CA test. Figure 5c displays the change in CL activity following the CA test as well as the corresponding change in TOF. The activity loss and TOF drop for NO-I were determined to be around 50%. For C–I, there was a small decrease in activity and TOF (around 5%). However, activity and TOF increased by around 10 and 20% for Mix-I and I–C, respectively. The change in total polarization resistance and ECSA is displayed in Figure 5d. Figure S9 shows EIS spectra at 1.55 V following a 60-step CA measurement. No-I showed a considerable increase in the overall polarization resistance and a significant decrease in the ECSA, as predicted. The CLs containing Nafion ionomer, on the other hand, showed a drop in total polarization resistance and an increase in ECSA, with I–C showing the most significant change. The changes in EDL capacitance, TOF, ECSA, and *R*_ct_ + *R*_1_ for all CLs are consistent with their activity loss or increase. The use of Nafion as a binder in CL considerably increases catalyst particle connectivity and prevents CL detachment during stability testing. However, the addition of an excess ionomer has the potential to reduce the number of active catalytic sites, resulting in mass transfer loss. The chemical and structural reconstruction of the EDL determines how the CLs change their activity following the stability test.

**Figure 6:**
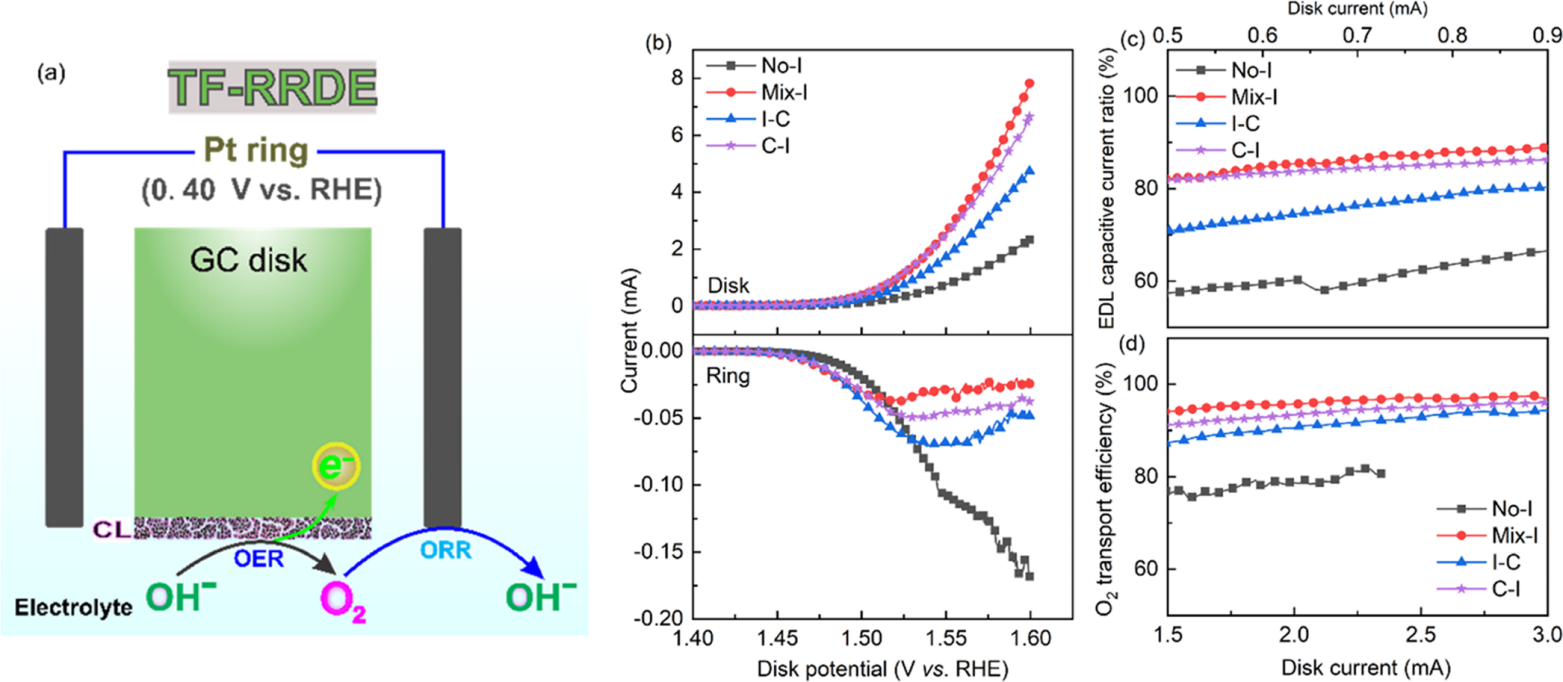
TF-RRDE evaluation of the effects of ionomer loading mode. (a) Schematic illustration of species transport behaviors in the TF-RRDE configuration. (b) RRDE voltammetry at the disk electrode (at a rotating rate of 2500 rpm and a scan rate of 1 mV s^–1^) and ring electrode (held at 0.40 V vs RHE). (c) EDL capacitive current ratio and (d) O_2_ transport efficiency as a function of disk current.

The catalyst activity for the OER is determined by effective charge transfer and transport of the O_2_ in the EDL. TF-RRDE voltammetry was used to quantify the contribution of various transport properties. Figure 6a illustrates a schematic of the TF-RRDE system. The disk potential was swept from 1.375 to 1.60 V at a scan rate of 2 mV s^–1^ during TF-RRDE voltammetry, and O_2_ generated at the disk was detected at the ring electrode fixed at 0.4 V to reduce O_2_ molecules. The faradaic mechanism might encompass an OER via a two-electron oxidation pathway, leading to the creation of an HO_2_^–^ intermediate. To investigate this phenomenon, the ring potential was set to 1.4 V to facilitate the oxidation of HO_2_^–.^^24,25,58^ In addition, We have also opted for a potential of 1.1 V, which is sufficiently elevated to facilitate the oxidation of H_2_O_2_ (in the diffusion-limited range), yet remains below the thermodynamic potential required for O_2_ evolution.^43^Figure S10 reveals negligible ring current detection in both cases. This result affirms that the electrocatalytic OER facilitated by the studied IrO_2_-based catalyst layers adheres to a four-electron transfer mechanism. Our previous study showed that oxygen generated during OER can be detected at the ring electrode by TF-RRDE in the low overpotential region, allowing the contribution of EDL charging to be precisely monitored.^25^ Faradaic OER produces the majority of the total current generated in the high potential zone (mixed kinetic-diffusion region). Therefore, the O_2_ transport efficiency can be estimated by subtracting the EDL charging current from the total disk current.

Figure 6b presents the TF-RRDE voltammetry results of various CLs. As the disk potential was scanned positively and reached the onset potential (1.4 V), the ORR current at the ring electrode began to grow, indicating the successful reduction of the O_2_ generated at the disk. The ring current reached a maximum and then decreased, forming a plateau as the disk potential was scanned to a higher potential zone. This indicates mass transport limitation at the ring due to the transported O_2_ from the disk. This observation suggests the presence of an O_2_ bubble effect, indicating that the majority of the generated O_2_ exited the disk electrode and entered the electrolyte solution without being reduced at the ring electrode.^25^ The CLs with the Nafion ionomer showed a smaller ring current than No-I. In the case of No-I, the OER proceeds at a slower rate, leading to a gradual release of oxygen from the disk electrode. Therefore, oxygen can be effectively detected by the ring electrode, resulting in a higher ring current compared with the other catalyst layers with Nafion. According to our previous study, the RRDE data were further processed to obtain the EDL capacitive current ratio and oxygen transport efficiency, which are presented in Figure 6c.^25^ When CLs containing Nafion were compared with those without the ionomer (No-I), the EDL capacitive current ratio was found to be substantially higher for the CLs containing Nafion. Compared to I–C, Mix-I and C–I had a comparable or greater EDL capacitive current ratio. In Figure S11, it is observed that adding the ionomer layer first and then casting the CL on top of it prevents the catalyst particles from having a proper connection and attachment to the electrode surface. In the FESEM image presented in Figure S11a, it becomes evident that the catalyst layer exhibits a loosely adhered connection with a Nafion layer that was cast before the catalyst layer itself. In Figure S11b, the catalyst layer demonstrates a reduction in particle density subsequent to the OER experiment. Furthermore, the O_2_ transport efficiency of the CLs follows the same trend as their EDL capacitive current ratio, demonstrating that the Nafion ionomer in CLs functions not only as a binder but also as a transport medium capable of achieving substantial OER activity. The impact of catalyst loading on the EDL capacitive current ratio and O_2_ transport efficiency has also been investigated and is shown in Figure S13. When the loading was increased to 0.24 mg cm^–2^, the EDL capacitive current ratio and oxygen transport efficiency for the CLs with a Nafion ionomer were drastically reduced. The overlapping catalyst surface rendered the catalytic active sites inaccessible to the reactants. The lower porosity of the CLs due to the higher loading caused a decrease in the O_2_ transport efficiency. It is worth highlighting that the magnitude of the EDL capacitive current ratio and the O_2_ transport efficiency of the catalyst layers will reduce if we consider a lower collection efficiency than the geometric collection efficiency (37.3%). However, the overall trend remains consistent and the comparisons among the four Nafion ionomer deposition modes are still valid.

**Figure 7:**
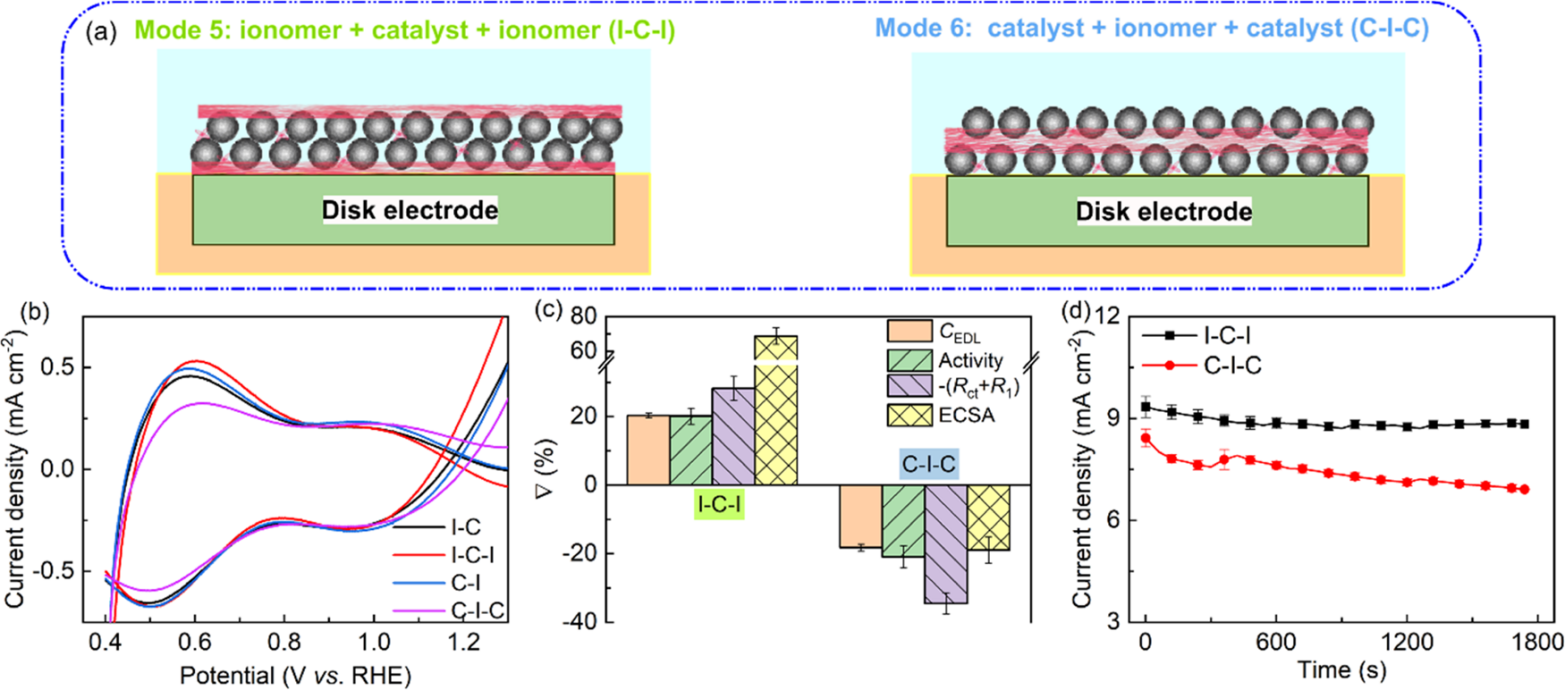
(a) Illustration of the coating modes I–C–I and C–I–C schematic for TF-RDE evaluation. (b) Modified CV curves at 50 mV s^–1^ to detect the pseudocapacitive behaviors. The shown current is the measured current subtracted by the EDL current at the same scan rate. (c) Performance alternation of I–C–I and C–I–C samples after the stability test when compared with I–C and C–I samples, respectively. (d) *I*–*t* curves at the high potential, 1.55 V, during CA measurement.

We investigated the influence of CL configuration on electrochemical parameters using a three-layer structure, namely, I–C–I and C–I–C, as schematically illustrated in Figure 7a,b. In Figure 7c, we assessed the role Nafion ionomer in building an efficient mass transport network by calculating the alternation percentage, ∇(%), between key parameters, ψ, obtained from TF-RDE tests, including *C*_EDL_, OER activity, −(*R*_ct_ + *R*_1_), and ECSA. The calculation is as follows:
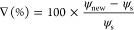
where ψ_new_ and ψ_s_ refer to the properties obtained from the new sample and the corresponding reference sample, respectively. The reference sample is either I–C (for the new sample, I–C–I) or C–I (for C–I–C). Raw experimental data of I–C–I and C–I–C are presented in Figures S14–S16, which were used to calculate the change in essential parameters illustrated in Figure 7c,7d. When comparing I–C–I and C–I–C to I–C and C–I, respectively, we observed that the overpotential at 10 mA cm^–2^ remains the same, as indicated by the LSV curves in Figure S14c. Apart from a significant positive change in *C*_EDL_ following the stability test, the major electrochemical parameters for I–C–I remain unaltered. Moreover, compared with I–C, the current densities during the stability test are higher. These findings suggest that adding Nafion to the top of the CL I–C not only serves as a binder to minimize catalyst loss but also enhances catalyst connection and accessibility to active catalyst sites. On the other hand, adding CL to the top of the ionomer layer in C–I makes the catalyst particles more prone to detachment during the OER test and reduces catalyst particle connection. As a result, during the stability test, C–I–C exhibited a negative shift in key electrochemical parameters and lower current densities. Although adjusting the drop-casting process can potentially improve the activity and stability of CLs, our results show that a catalyst ink containing well-dispersed Nafion ionomer produces the best CL with superior activity and stability for OER. This can be attributed to the homogeneous distribution of the ionomer in the CL. Furthermore, following the stability test, the OER activity of I–C increased more than that of Mix-I. This can be attributed to the cyclic transition of hydroxylated metal cations with various oxidation states, leading to reconstruction.^17,59^ Continuous EDL reconstruction appears to be advantageous for OH^–^ adsorption in Mix-I and I–C. Hence, achieving an optimal distribution of Nafion in the CL is crucial to ensure the required adhesion to GCE, efficient charge transport, and prevention of catalyst dissolution during the OER, resulting in increased activity and stability of the IrOx catalyst material.

## 4 Conclusions

In conclusion, this study investigated the role of Nafion in different modes of catalyst loading for the OER in an alkaline medium. The CLs containing Nafion ionomer exhibited significantly lower overpotential in the kinetic region (10 mA cm^–2^) compared to Nafion-free CLs (No-I). The turnover frequency of Mix-I was approximately twice as high as that of No-I. This indicates that the presence of Nafion not only improves CL adhesion but also enhances catalyst particle connection and intrinsic OER activity. However, a higher loading was found to negatively affect the EDL charging capacitance and the O_2_ transport efficiency of the CLs. By adding Nafion to catalyst inks, interfacial reversibility is improved, and anodic dissolution of IrO*_x_* is prevented, as supported by the investigation of EDL capacitive behavior. Additionally, achieving the proper distribution of Nafion ionomer in the CL can further enhance OER catalytic activity. The findings of this study underscore the indispensable role of Nafion ionomer and its crucial interaction with the catalyst through various processing modes for OER. These combined results offer fresh insights into the optimal electrode design for electrolysis cells in hydrogen technology.

## Supporting Information Available

Additional information on tables of impedance fitting parameters, FESEM images of the catalyst layers, and figures of electrochemical results (PDF)
