# Supplementary material for: Indispensable Nafion Ionomer for High-Efficiency and Stable Oxygen Evolution Reaction in Alkaline Media
Source: ACS Appl Mater Interfaces. 2023 Nov 21;15(48):55559–69. doi: 10.1021/acsami.3c08377 (PMC10711702; doi:10.1021/acsami.3c08377)
Supplement: Supplementary file 1 — am3c08377_si_001.pdf [file am3c08377_si_001.pdf]

Supporting information for

Indispensable Nafion ionomer for high-efficiency and stable oxygen evolution reaction in alkaline media

Nitul Kakati,[1] Lawrence Anderson,[1] Guangfu Li,[,1,2] Desiree Mae Sua-an,[1,3] Ayon Karmakar[1], Joey D. Ocon,[3] and Po-Ya Abel Chuang[*,1]*

[1]Department of Mechanical Engineering, University of California Merced, California 95343, United States

[2]Foshan Xianhu Laboratory of the Advanced Energy Science and Technology Guangdong Laboratory, Xianhu Hydrogen Valley, Foshan, 528200, China

[3]Laboratory of Electrochemical Engineering, Department of Chemical Engineering, University of the Philippines Diliman, Quezon City 1101, Philippines

*Corresponding authors: abel.chuang@ucmerced.edu.

*Co-Corresponding Author: liguangfu@xhlab.cn

Table S1. Impedance fitting parameters at 1.55 V and the calculated C^*_{EDL} and ECSA.

Sample[a]	L (e-6 H cm^{-2})	R_Ω (Ω cm^2)	R_{ct} (Ω cm^2)	CPE$_1$		R_1 (Ω cm^2)	CPE$_2$		C^*_{EDL} (mF cm^{-2})	ECSA (g m^{-2})
				Y_o (mF cm^{-2} s^{n-1})	n_1		Y_o (mF cm^{-2} s^{n-1})	n_2		
NO-I	7.36	0.30	5.53	2.30	0.885	1.86	0.78	0.686	2.18	45.37
NO-I_S	5.55	0.36	7.50	2.04	0.826	3.29	0.20	0.863	1.91	39.70
MIX-I	7.02	0.43	2.82	3.64	0.906	0.20	675	0.377	3.77	78.47
MIX-I_S	3.07	0.43	2.15	3.71	0.957	0.44	98.5	0.595	3.76	78.39
I-C	6.16	0.41	4.97	2.09	0.926	0.042	2760.	0.180	2.05	42.65
I-C_S	3.83	0.42	3.81	3.04	0.905	0.24	1530	0.333	3.08	64.23
C-I	8.80	0.44	3.08	3.46	0.918	0.073	4.97	0.767	3.55	73.95
C-I_S	7.99	0.41	2.56	3.47	0.951	0.19	41.74	0.368	3.51	73.18
C-I-C	8.19	0.51	3.89	2.81	0.913	0.13	4.46	0.782	2.88	59.90
C-I-C_S	6.51	0.44	3.71	3.02	0.934	0.11	15.4	0.658	3.06	63.80
I-C-I	7.01	0.44	3.81	3.04	0.923	0.087	56.5	0.602	3.09	64.41
I-C-I_S	5.21	0.42	2.87	3.29	0.935	0.20	395.8	0.412	3.34	69.49

[a]Sample with postfix "_S" is the sample after stability tests.

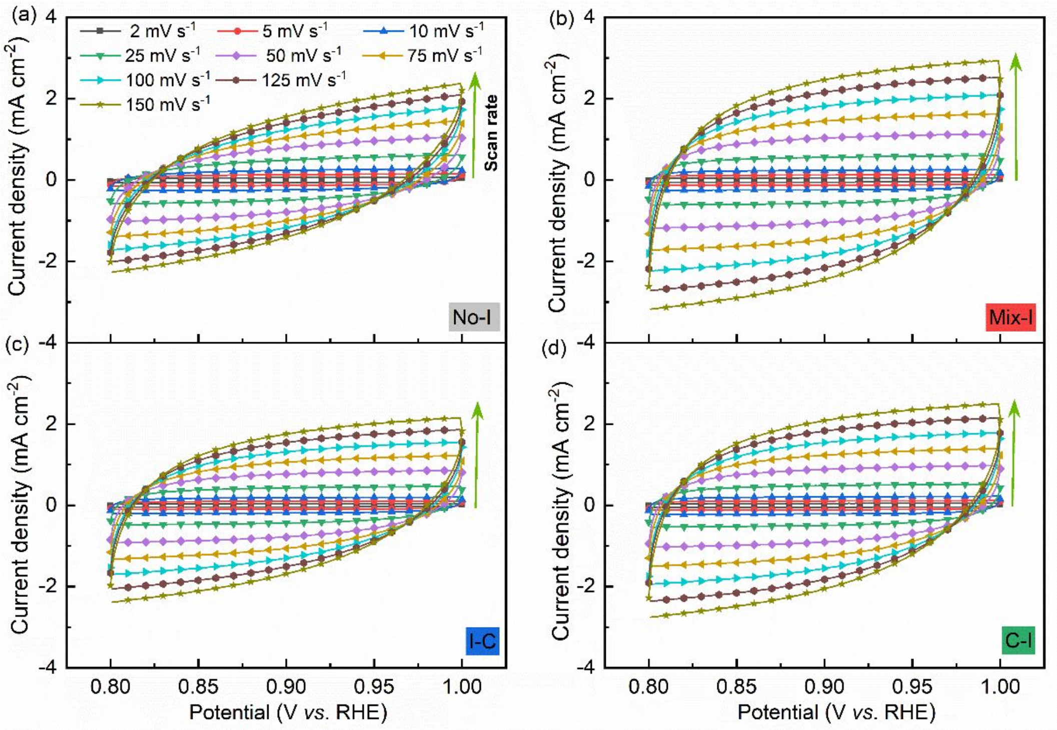

Figure S1. CV curves between 0.8-1.0 V with various scan rates.

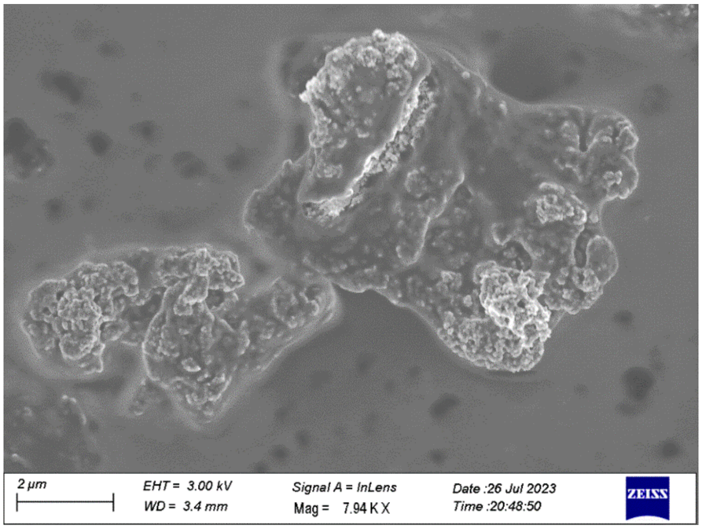

Fig. S2. An SEM image showing the catalyst particles covered by the Nafion ionomer in C-I mode of CL loading.

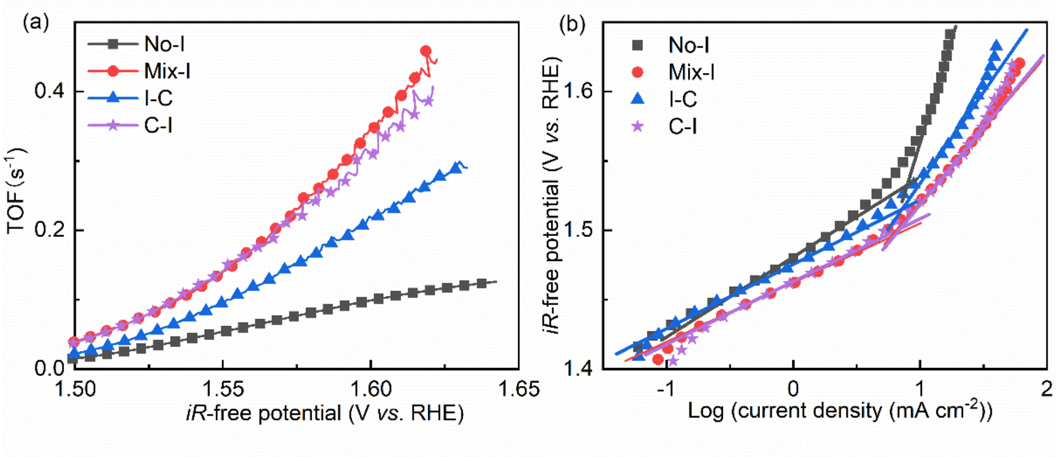

Figure S3. (a) TOF plots as a function of *iR*-free potential, and (b) Tafel plots, symbols - raw data; lines – linearly fitted results.

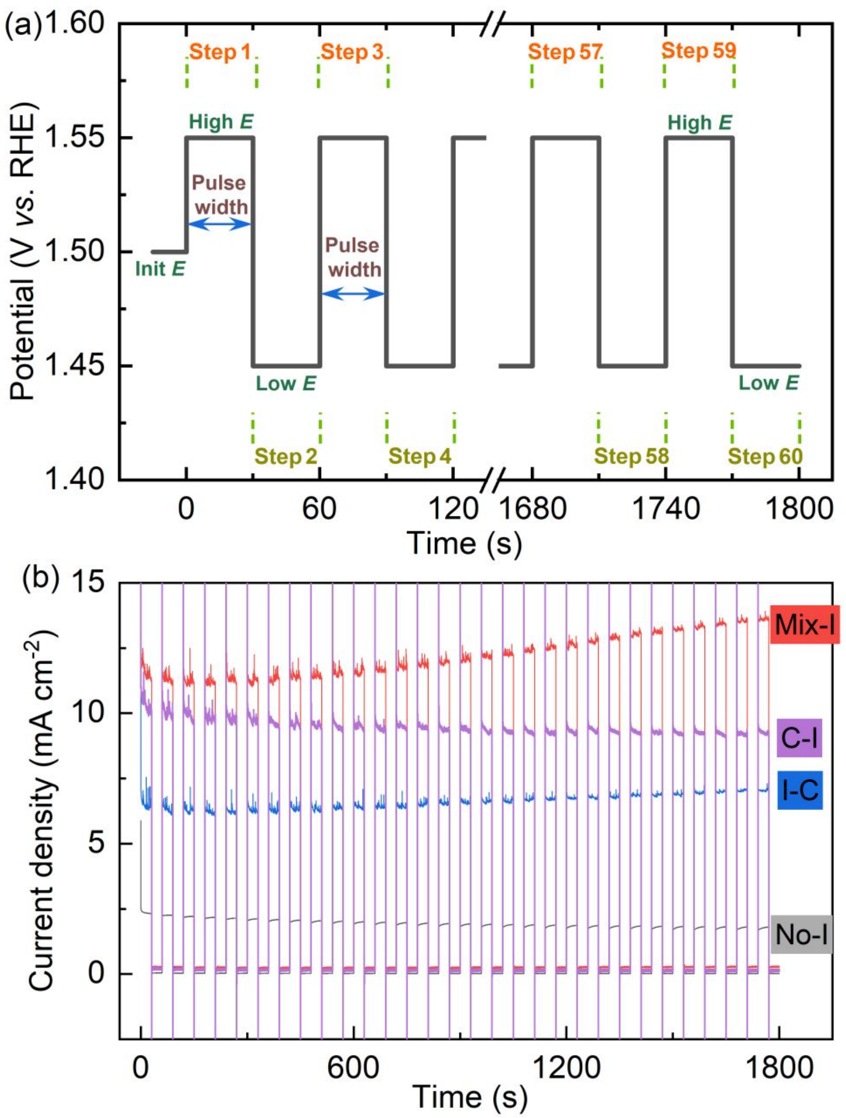

Figure S4. (a) Diagram of chronoamperometry (CA) measurement with primary technical parameters and (b)the corresponding *I*-*t* plots during the whole measurement.

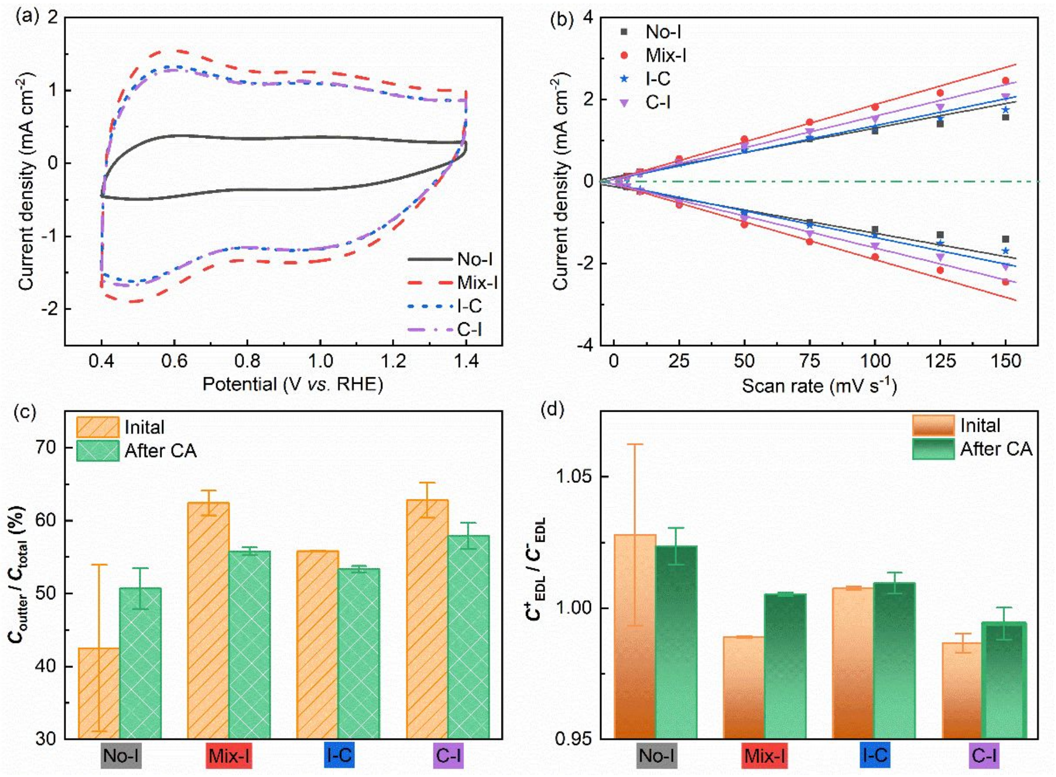

Figure S5. After 60-step CA measurements, investigation of solid-state pseudocapacitive and EDL capacitive behaviors. (a) CV curves measured at 50 mV s^{-1} and (b) the anodic (above zero) and cathodic (below zero) EDL capacitive current density (at 0.9 V) as a function of scan rate, symbols - raw data; lines – linear simulation results in the scan rate range of 2-100 mV s^{-1}. And the ratios of outer capacitance to inner capacitance and (d) anodic EDL capacitance to cathodic EDL capacitance.

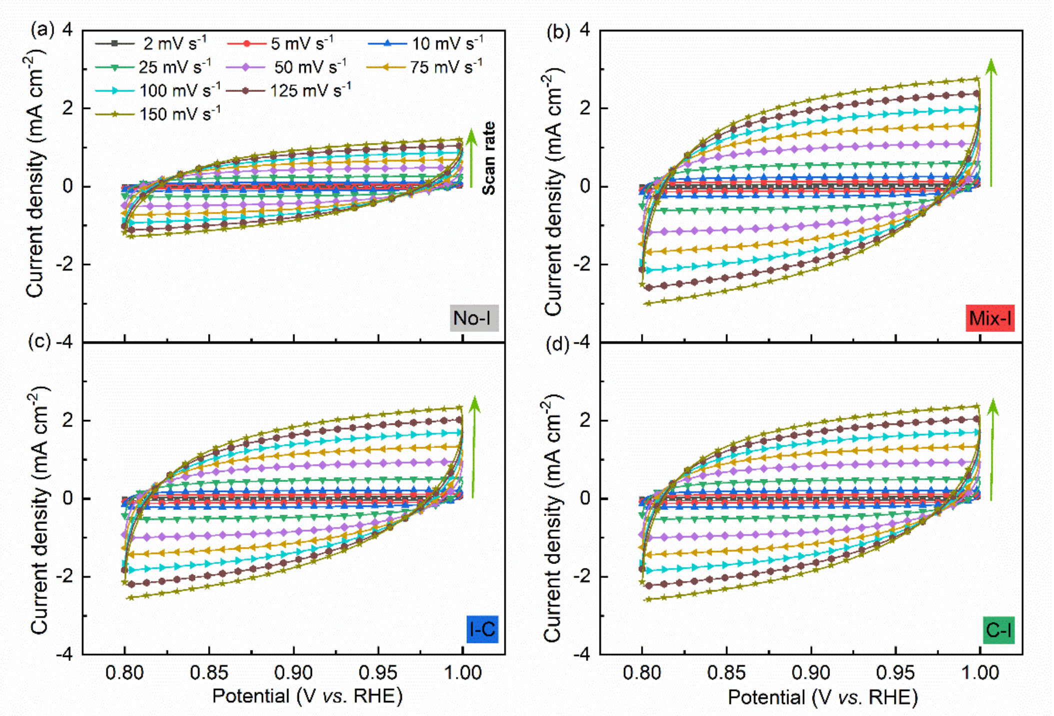

Figure S6. CV curves with various scan rate after 60-step CA measurements.

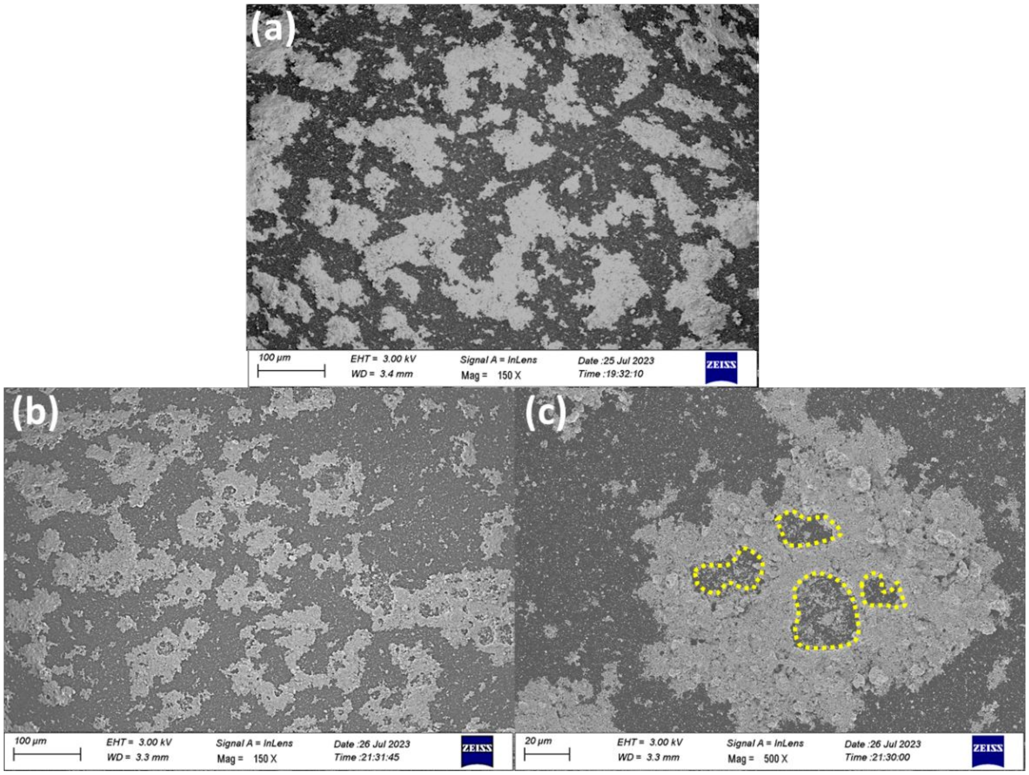

Figure S7. FESEM images of the No-I Catalyst Layer showing (a) Fresh CL before stability test and (b, c) CL after stability test.

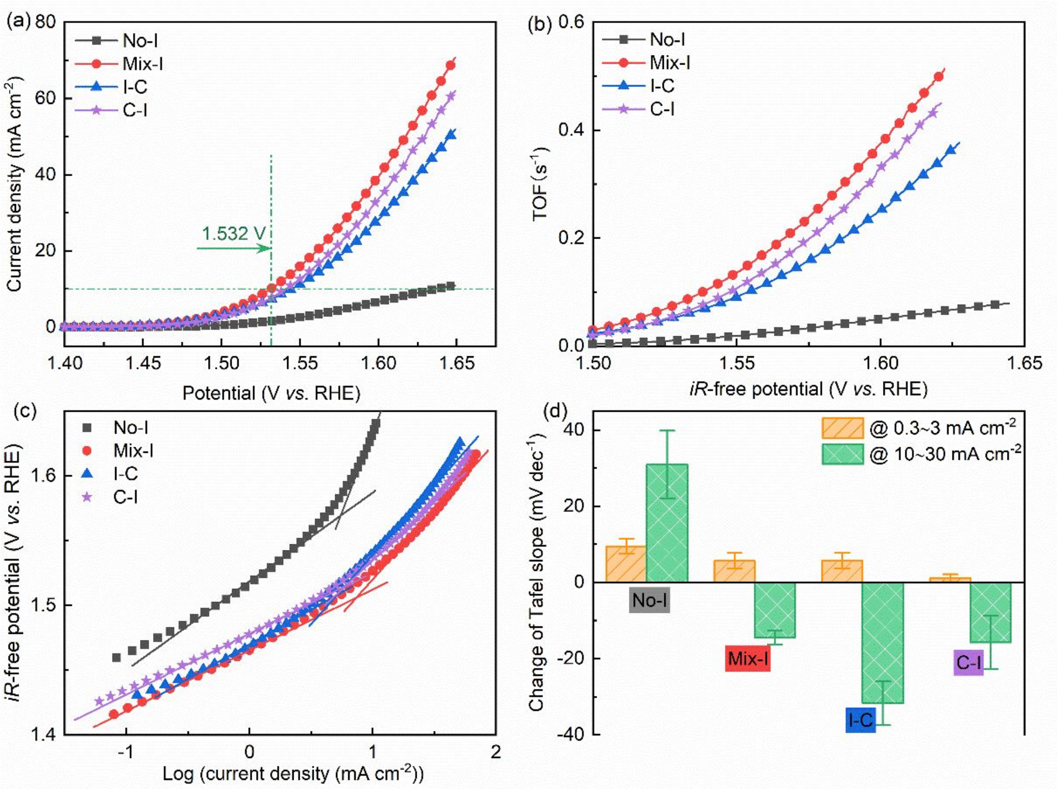

Figure S8. (a) LSV plots at 1 mV s-1, (b) TOF plots as function of iR-free potential, (c) Tafel plots, symbols - raw data; lines – linearly fitted results. And (d) change of Tafel slopes in the range of low and high current density after 60-step CA measurements.

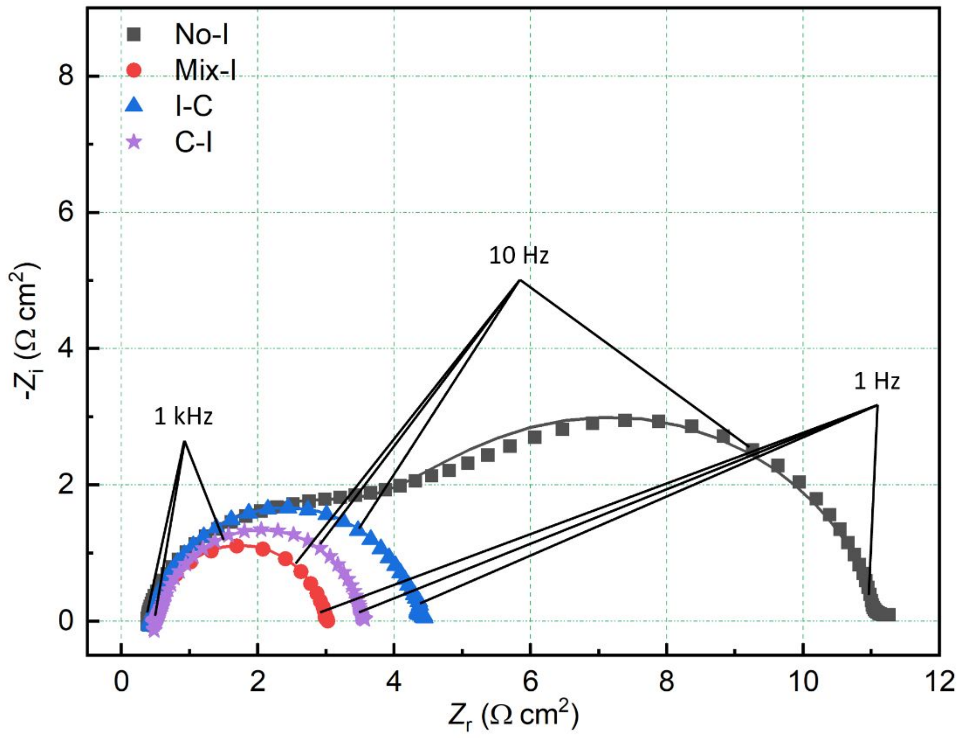

Figure S9. EIS spectra at 1.55 V after 60-step CA measurement. Symbols - raw data; lines - simulation results.

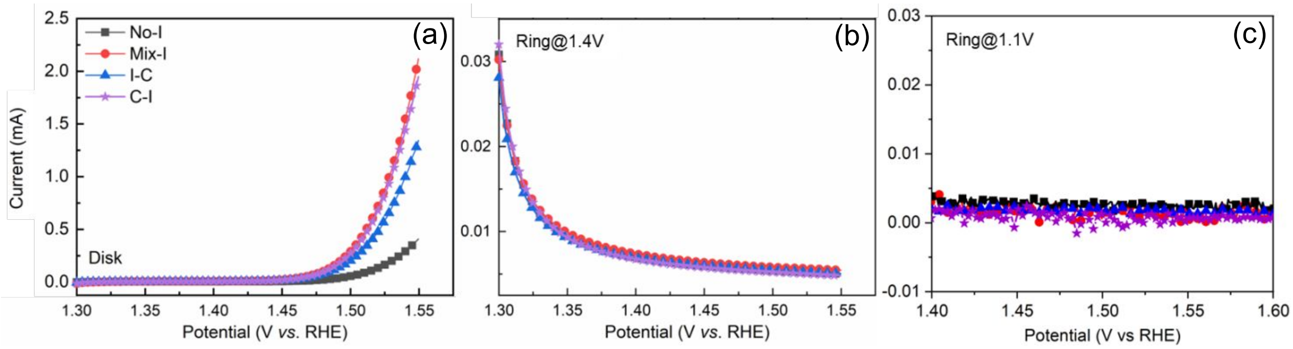

Figure S10. (a) TF-RRDE voltammogram at disk electrode (with a rotating rate of 2500 rpm and a scan rate of 1 mV s^{-1}) and (b, c) ring electrode (holding at 1.40 V and 1.10 V vs. RHE).

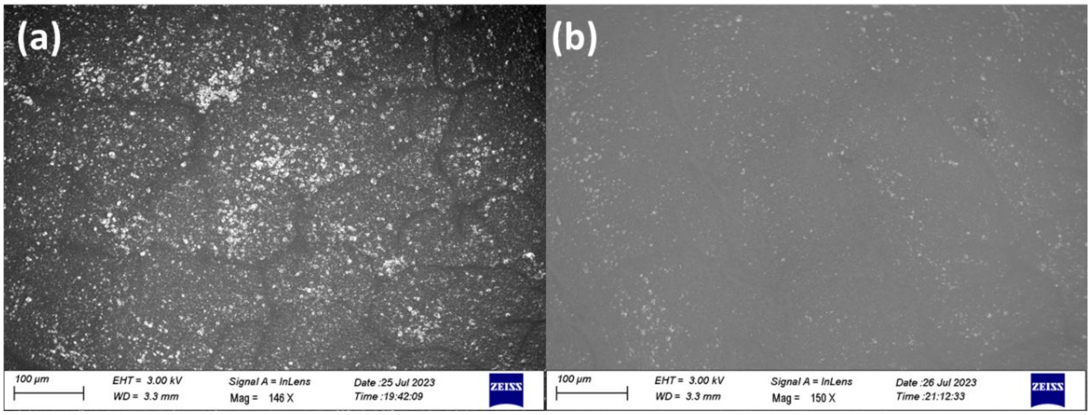

Figure S11. FESEM images of the I-C catalyst layer (a) before and (b) after the OER experiments.

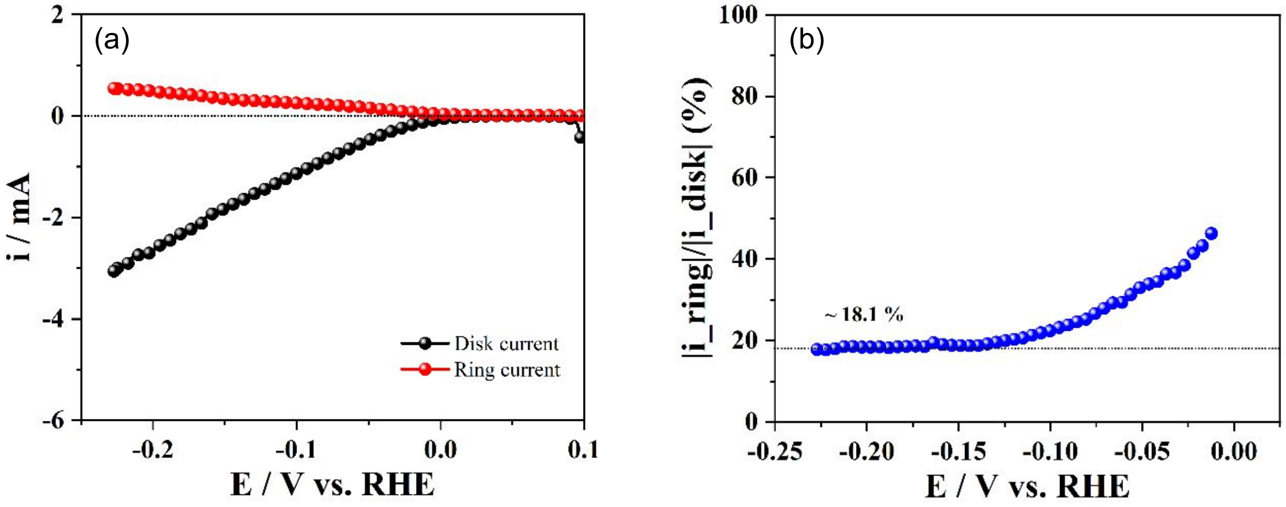

Figure S12. (a) RRDE method to carry out HER with Pt/C at disk and HOR on the ring in 0.1 M KOH (b) The collection efficiency calculated from measured disk and ring current.

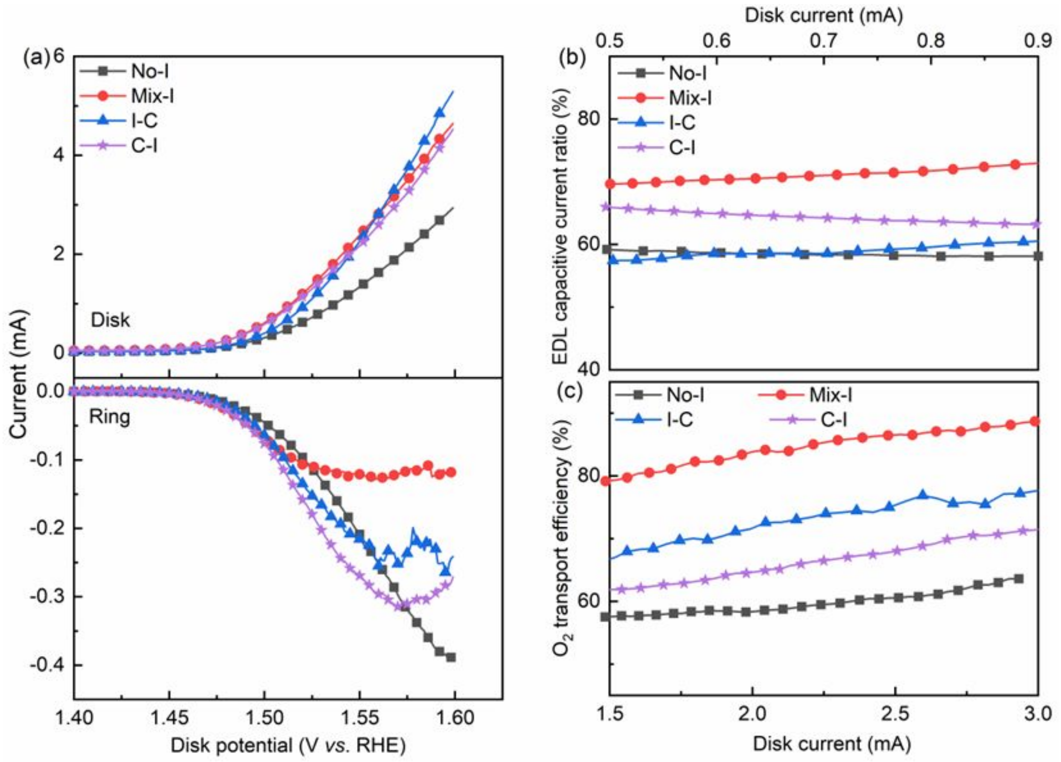

Figure S13. RRDE evaluation with high catalyst loading in 1 M KOH. (a) RRDE voltammetry at disk electrode (with 0.24 mg cm^{-2} catalyst loading and a scan rate of 1 mV s^{-1}) and ring electrode (holding at 0.40 V for ORR). (b) EDL capacitive current ratio and (c) O_2 transport efficiency as a function of disk current.

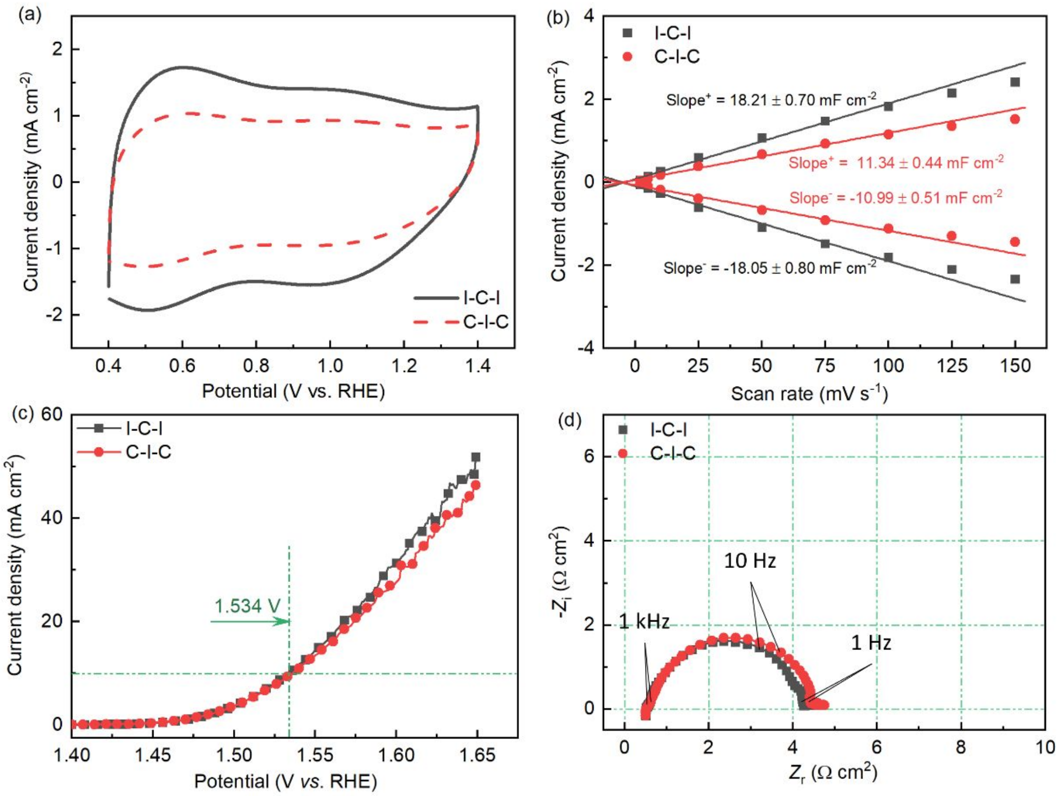

Figure S14. Raw experimental results of I-C-I and C-I-C samples on the TF-RDE configuration using 6 M KOH. (a) CV curves at 50 mV s^{-1}, (b) the anodic (above zero) and cathodic (below zero) EDL capacitive current density (at 0.9 V) as a function of scan rate, symbols - raw data; lines – linear simulation results between 2-100 mV s^{-1}. (c) LSV plots at 1 mV s-1, and (d) EIS spectra at 1.5 V, Symbols - raw data; lines - simulation results.

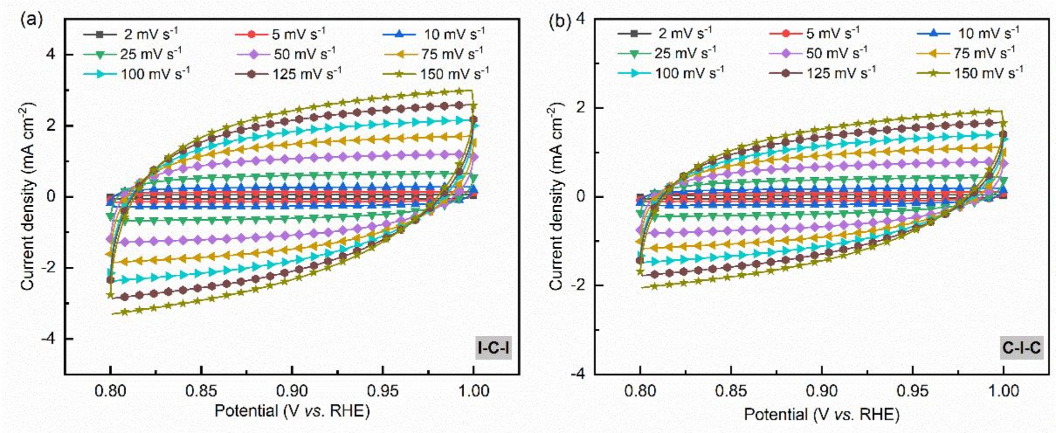

Figure S15. CV curves of I-C-I and C-I-C samples with various scan rates.

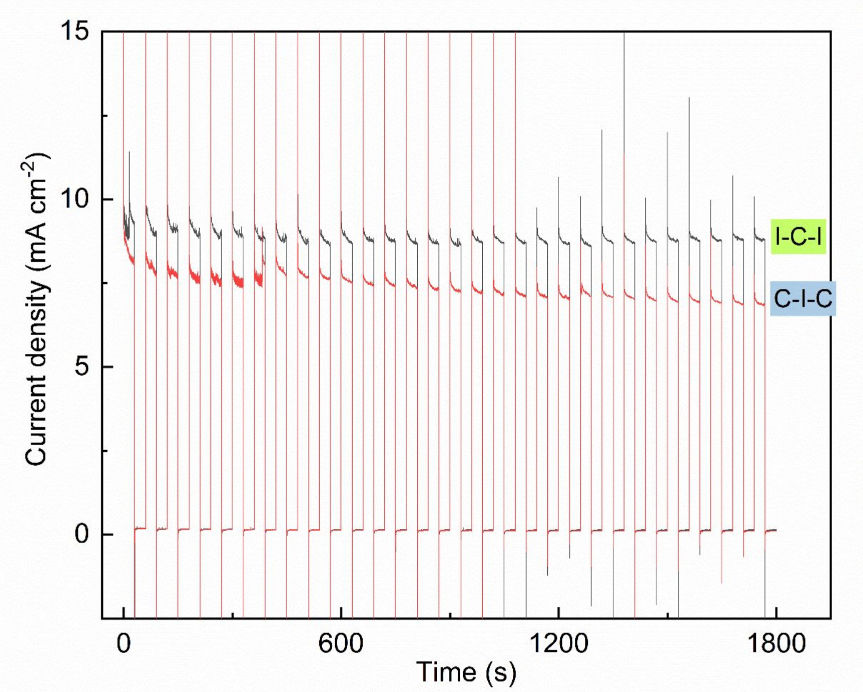

Figure S16. 60-step CA measurement of I-C-I and C-I-C samples at low and high potential.
